# Bradycardia and hypotension associated with trigeminocardiac reflex during orthognathic surgery: two case reports

**DOI:** 10.1097/MS9.0000000000003408

**Published:** 2025-05-29

**Authors:** Reika Hasegawa, Shogo Hasegawa, Hitoshi Miyachi, Satoshi Watanabe, Sanako Nakaya, Mitsuo Goto

**Affiliations:** a Department of Oral and Maxillofacial Surgery, School of Dentistry, Aichi Gakuin University, Nagoya-shi, Aichi-ken, Japan

**Keywords:** bradycardia, case report, hypotension, orthognathic surgery, trigeminal nerve stimulation, trigeminocardiac reflex

## Abstract

**Introduction and importance:**

The trigeminocardiac reflex (TCR) is an uncommon but significant complication that can arise during orthognathic surgery.

**Case Presentation:**

We present two cases of TCR-induced bradycardia and hypotension during such procedures. In the first case, bradycardia was noted following a controlled downward fracture of the maxilla. In the second case, both bradycardia and hypotension occurred while the left mandibular ramus was split with a separator.

**Clinical Discussion:**

In both instances, interruption of the surgical procedure led to normalization of heart rate and blood pressure, indicating that TCR was likely triggered by stimulation of the second and third branches of the trigeminal nerve. Moreover, to the best of our knowledge, this is the first reported case of TCR during Le Fort I osteotomy in Japan.

**Conclusion:**

Given that TCR can potentially lead to cardiac arrest, surgeons must anticipate this reflex and communicate closely with the anesthesiologist to ensure prompt management.

## Introduction

The trigeminocardiac reflex (TCR) is a physiologic response involving bradycardia, hypotension, and potential cardiac arrest triggered by stimulation of any branch of the trigeminal nerve. While TCR commonly occurs in ophthalmologic procedures—particularly during strabismus surgery—it is an infrequent complication of maxillofacial surgery^[^1,2^]^. In Japan, the descending palatine artery is typically preserved during Le Fort I osteotomy, which may explain the lack of reported cases on TCR during maxillary down-fracture. We present two cases of TCR during orthognathic surgery under general anesthesia.

**HIGHLIGHTS**

- The trigeminocardiac reflex (TCR) can occur during orthognathic surgery.
- If TCR occurs, immediate cessation of the surgical stimulus is crucial.
- Both surgeons and anesthesiologists need to keep in mind that tcr can occur during orthognathic surgery.

## Case presentation

### Case 1

A 39-year-old woman (weight: 56 kg; height: 158.4 cm) with mandibular protrusion was scheduled for Le Fort I osteotomy and bilateral sagittal split ramus osteotomy (BSSRO) under general anesthesia. The patient had no history of anesthesia exposure or any comorbidities. Physical examination and preoperative assessment, including chest radiography, electrocardiography (ECG), and blood chemistry, were normal. Prior to entering the operating room, her blood pressure and heart rate were recorded at 151/81 mmHg and 91 beats/min, respectively.

Anesthesia was induced with intravenous propofol, remifentanil, and rocuronium, followed by uneventful nasoendotracheal intubation. Maintenance anesthesia included propofol, an oxygen/air mixture to achieve a bispectral index (BIS) of 40–60, and remifentanil. Ventilation was adjusted to maintain an end-tidal carbon dioxide concentration (EtCO_2_) of 30–35 torr. Continuous monitoring of ECG, pulse oximetry, EtCO_2_, and arterial blood pressure was performed and recorded at 2.5-minute intervals.

**Figure 1.**
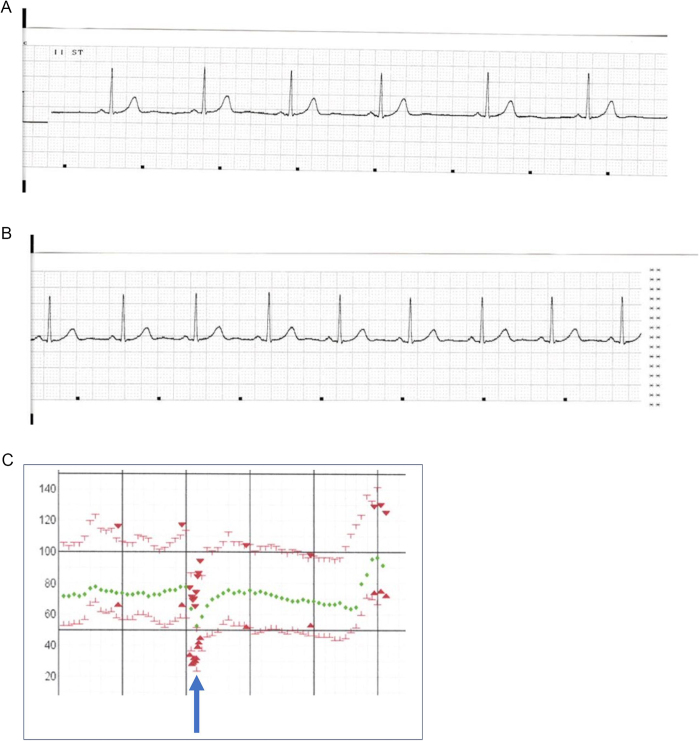
Electrocardiogram and anesthesia record. (A) Heart rate decreases to 46 beats/min in Case 1. (B) Recovery to approximately 60 beats/min in Case 1. (C) Blood pressure and heart rate are reduced to 54/25 mmHg and 52 beats/min, respectively, (blue arrow) in Case 2. ┸ ┰: ABP (Arterial Blood Pressure), ▼▲: BP, ◆: HR.

The Le Fort I osteotomy began 43 min after anesthesia induction. Initially, ECG findings were unremarkable, and heart rate was stable at 60–70 beats/min. However, during the down-fracture of the maxilla, bradycardia abruptly developed, with the heart rate decreasing to 46 beats/min (Fig. 1A and 1B). The surgeon was immediately informed, and the procedure was ceased. The patient’s heart rate subsequently recovered to approximately 60 beats/min. After 5 minutes, surgery resumed with supplemental local anesthesia and proceeded without further arrhythmia or bradycardia.

The total operation time was 5 h 16 min, and the anesthesia duration was 6 h 17 min. At discharge, blood pressure was 140/72 mmHg, and heart rate was 94 beats/min. The maxilla was repositioned with an elevation of 5 mm near the right molar and 2 mm near the left molar. The mandible was repositioned bilaterally.

### Case 2

A 26-year-old male (weight: 76.5 kg, height: 185.2 cm) with mandibular protrusion was scheduled for Le Fort I osteotomy and BSSRO under general anesthesia. The patient had no history of anesthesia exposure or comorbidities. Physical examination findings were normal, and preoperative investigations, including chest radiography, ECG, and blood chemistry, were within normal limits. He had a blood pressure of 136/78 mmHg and a heart rate of 83 beats/min before entering the operating room were and, respectively.

Anesthesia was induced with intravenous propofol, remifentanil, and rocuronium, followed by successful nasoendotracheal intubation. During surgery, anesthesia was maintained using sevoflurane in an oxygen/air mixture to achieve a BIS of 40–60 with remifentanil and fentanyl. Ventilation was adjusted to maintain an EtCO_2_ of 30–35 torr. ECG, pulse oximetry, EtCO_2_, and arterial blood pressure were measured continuously and recorded every 2.5 minutes.

Le Fort I osteotomy began 55 minutes after anesthesia induction. Initially, there were no ECG abnormalities, and the heart rate was stable at 60–70 beats/min. The Le Fort I osteotomy proceeded uneventfully. Afterward, BSSRO was initiated with right mandibular splitting, maintaining stable circulatory dynamics. During the left osteotomy, a preoperative autologous blood transfusion was started.

Approximately 20 minutes after the start of autotransfusion, the patient experienced an abrupt decline in the heart rate and blood pressure, measuring 52 beats/min and 54/25 mmHg, respectively, during the splitting of the left mandibular ramus with a separator (Fig. 1C). Autotransfusion was immediately paused, and ephedrine hydrochloride was administered. Heart rate and blood pressure subsequently improved to 67 beats/min and 102/46 mmHg, respectively. Surgery was resumed and concluded without further intraoperative complications.

The total operation time was 5 hours 12 minutes, with anesthesia lasting 6 hours 28 minutes. At discharge, BP was 124/74 mmHg, and the heart rate was 94 beats/min. The maxilla was elevated by 3 mm with a 3 mm forward advancement, while the mandible was repositioned by 13 mm on the right side and 8.5 mm on the left side.

In both cases, the surgeries were performed by surgeons with more than 10 years of experience who are certified as specialists by the Japanese Society of Oral and Maxillofacial Surgeons. Postoperative panoramic radiographs were taken to confirm the absence of any abnormal fractures.

## Discussion

TCR is defined as a reduction in heart rate and mean arterial pressure of more than 20% from baseline, dysrhythmias, or sinus arrest triggered by surgical manipulation near any branch of the trigeminal nerve.^[^3-6^]^ TCR, also known as the trigeminal vagus reflex, is a parasympathetic reflex with the trigeminal nerve serving as the afferent pathway and the vagus nerve as the efferent pathway. Stimulation of the trigeminal region can induce sudden bradycardia, arrhythmia, or sinus arrest[7]. Initially described in 1908 as the oculocardiac reflex, related to orbital and periorbital surgery[8], the scope of TCR was broadened in 1988 by Shelly *et al*, who refined the term to encompass reflexes triggered by stimulation of any branch of the trigeminal nerve or the main nerve trunk^[^3,9,10^]^.

**Figure 2.**
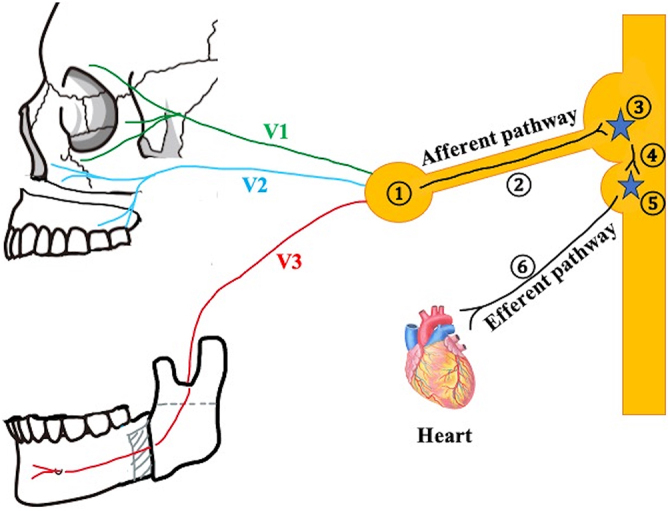
Trigeminocardiac reflex pathway. TCR occurs when sensory branches of the trigeminal nerve transmit signals from the Gasserian ganglion to the sensory nucleus of the trigeminal nerve on the floor of the fourth ventricle (afferent pathway). These signals are then relayed to the vagus motor nucleus via short nerves and directed to the myocardium via the cardiac branches of the vagus nerve (efferent pathway). For example, traction of the posterior superior alveolar nerve or the greater palatine nerve during Le Fort I osteotomy and traction of the inferior alveolar nerve during SSRO can act as triggers. V1: ophthalmic nerve; V2: maxillary nerve; V3: mandibular nerve. 1. Gasserian ganglion; 2. Trigeminal nerve; 3. Sensory nucleus of the trigeminal nerve; 4. Short internuncial fibers; 5. Motor nucleus of the vagus nerve; 6. Cardiac branch of the vagus nerve.

TCR occurs when sensory branches of the trigeminal nerve transmit signals from the Gasserian ganglion to the sensory nucleus of the trigeminal nerve on the floor of the fourth ventricle (afferent pathway). These signals are then relayed to the vagus motor nucleus via short nerves and directed to the myocardium via the cardiac branches of the vagus nerve (efferent pathway) (Fig. 2). ^[^3,11^]^

In our cases, we hypothesize that the stretching of the greater palatine or posterior superior alveolar nerves may have triggered TCR in Case 1; in Case 2, the involvement of the mandibular nerve could have acted as the stimulus. In Case 2, there was hypotension and bradycardia during the transfusion. However, considering that the blood was returned very slowly and that it was an autologous transfusion, we believed the possibility of hypotension due to a transfusion reaction was low.

TCR is a well-recognized complication in ophthalmologic surgery, occurring in approximately 25% of blepharoplasty procedures and 32%–90% of strabismus surgeries[1]. However, its incidence in maxillofacial surgery is relatively low, with a reported frequency of 1.6%[12]. TCR has been associated with procedures such as zygomatic fracture reconstruction, orthognathic surgery, temporomandibular arthroscopy, extraction of overfilled maxillary median teeth, and extraction of filled mandibular wisdom teeth^[^1,3,7,13,14^]^. Six cases of TCR during orthognathic surgery have been documented in Japan (Table 1).

**Table 1:** Trigeminocardiac reflex during orthognathic surgery in Japan

Authors	Age	Sex	Surgery	Trigger surgical procedure	Clinical feature	HR (beats/min)	Treatment
Sanuki et al., 2009[15]	31	F	BSSRO	Soft tissue dissection	Sinus arrest	30–35	Interruption of the surgery, administration of anticholinergic drugs
Miyamoto *et al*, 2012[13]	18	F	Le Fort I, BSSRO, genioplasty	Suturing the mandibular mucoperiosteal flap	Bradycardia	12	Interruption of the surgery, cardiac massage, administration of anticholinergic drugs
Kumasaka *et al*, 2013[16]	18	F	Le Fort I, BSSRO	Placing a retractor along the medial aspect of the mandibular ramus	Bradycardia, hypotension	Approximately 30	Interruption of the surgery, administration of anticholinergic drugs
	39	M	BSSRO	Placing a retractor along the medial aspect of the mandibular ramus	Bradycardia	Approximately 30	Interruption of the surgery
Wakasugi *et al*, 2013[17]	21	M	Le Fort I, BSSRO	Manipulation of the mandible	Bradycardia, sinus arrest	45	Interruption of the surgery
Sugiyama *et al*, 2020[18]	31	F	Le Fort I, BSSRO	Splitting of the mandibular ramus with a separator	Bradycardia	29	Interruption of the surgery, administration of anticholinergic drugs
Our cases, 2023	39	F	Le Fort I, BSSRO	Down fracture	Bradycardia	46	Interruption of the surgery
	25	M	Le Fort I, BSSRO	Splitting of the mandibular ramus with a separator	Bradycardia, hypotension	52	Interruption of the surgery, administration of ephedrine

BSSRO, bilateral sagittal split ramus osteotomy; F, Female; M, Male.

Risk factors for the TCR include a history of cardiac disease, hypercapnia, hypoxemia, shallow anesthesia, vagal dominance (high vagal tone at rest), narcotic use, and preoperative beta-blocker or calcium channel blocker therapy^[^1,4,16^]^. Additionally, remifentanil is known to strongly suppress the parasympathetic nervous system^[^15,19,20^]^, while propofol has sympathoinhibitory effects, promoting relative parasympathetic dominance and bradycardia. In cases of TCR, continuous administration of remifentanil and propofol, combined with nociceptive stimulation of the trigeminal nerve, may contribute to reflex onset.

Prevention of TCR requires close collaboration between the surgeon and anesthesiologist. Clear communication from the surgeon during perineural manipulation and maintaining adequate anesthesia depth by the anesthesiologist are key preventive strategies.

Additionally, sufficient infiltration anesthesia and nerve blocks, as well as avoiding abrupt manipulation, may help prevent the reflex^[^17,19,21^]^. Previous studies report have shown that when comparing groups with and without nerve blocks, the mean heart rate reduction during the splitting and setback steps of SSRO was significantly greater in the non-blocked group compared to the blocked group[2]. While TCR can be reduced with nerve block, complete prevention of the reflex may not be achievable^[^2,19^]^.

If sudden bradycardia or asystole occurs, immediate cessation of the surgical stimulus is crucial. In most cases, stopping the stimulus allows the patient to regain sinus rhythm. Anticholinergics and cardiac massage may also be employed if necessary^[^1,13,22^]^.

## Conclusion

In summary, TCR is a potentially life-threatening complication that can result in severe bradycardia, cardiac arrest, and even mortality during maxillofacial surgery. Although uncommon, surgeons must remain vigilant about the possibility of this reflex occurring. Early recognition and prompt intervention, including cessation of the surgical stimulus and administering appropriate pharmacologic support, are essential to prevent adverse outcomes.

## Data Availability

The data supporting this study’s findings are available from the corresponding author upon reasonable request.

## Methods

The case has been reported in line with the surgical case report (SCARE) 2023 criteria[23].
